# Epigenetic analysis in placentas from sickle cell disease patients reveals a hypermethylation profile

**DOI:** 10.1371/journal.pone.0274762

**Published:** 2022-09-21

**Authors:** Gislene Pereira Gil, Galina Ananina, Mariana Maschietto, Sheila Coelho Soares Lima, Sueli Matilde da Silva Costa, Leticia de Carvalho Baptista, Mirta Tomie Ito, Fernando Ferreira Costa, Maria Laura Costa, Mônica Barbosa de Melo

**Affiliations:** 1 Center for Molecular Biology and Genetic Engineering (CBMEG), University of Campinas-UNICAMP, Campinas, São Paulo, Brazil; 2 Boldrini Children’s Center, Campinas, São Paulo, Brazil; 3 National Cancer Institute (INCA), Rio de Janeiro, Rio de Janeiro, Brazil; 4 Hematology and Hemotherapy Center, University of Campinas, Campinas, São Paulo, Brazil; 5 Department of Obstetrics and Gynecology, University of Campinas, Campinas, São Paulo, Brazil; Barts and The London School of Medicine and Dentistry Blizard Institute, UNITED KINGDOM

## Abstract

Pregnancy in Sickle Cell Disease (SCD) women is associated to increased risk of clinical and obstetrical complications. Placentas from SCD pregnancies can present increased abnormal findings, which may lead to placental insufficiency, favoring adverse perinatal outcome. These placental abnormalities are well known and reported, however little is known about the molecular mechanisms, such as epigenetics. Thus, our aim was to evaluate the DNA methylation profile in placentas from women with SCD (HbSS and HbSC genotypes), compared to uncomplicated controls (HbAA). We included in this study 11 pregnant women with HbSS, 11 with HbSC and 21 with HbAA genotypes. Illumina Methylation EPIC BeadChip was used to assess the whole placental DNA methylation. Pyrosequencing was used for array data validation and qRT-PCR was applied for gene expression analysis. Our results showed high frequency of hypermethylated CpGs sites in HbSS and HbSC groups with 73.5% and 76.2% respectively, when compared with the control group. Differentially methylated regions (DMRs) also showed an increased hypermethylation status for the HbSS (89%) and HbSC (86%) groups, when compared with the control group methylation data. DMRs were selected for methylation validation (4 DMRs-HbSS and 3 DMRs the HbSC groups) and after analyses three were validated in the HbSS group, and none in the HbSC group. The gene expression analysis showed differential expression for the *PTGFR* (-2.97-fold) and *GPR56* (3.0-fold) genes in the HbSS group, and for the *SPOCK1* (-2.40-fold) and *ADCY4* (1.80-fold) genes in the HbSC group. Taken together, these data strongly suggest that SCD (HbSS and HbSC genotypes) can alter placental DNA methylation and lead to gene expression changes. These changes possibly contribute to abnormal placental development and could impact in the clinical course, especially for the fetus, possibly leading to increased risk of abortion, fetal growth restriction (FGR), stillbirth, small for gestational age newborns and prematurity.

## Introduction

Sickle cell disease (SCD) comprehends a group of inherited disorders, characterized by the presence of hemoglobin S (HbS). HbS can be in the homozygous state (called sickle cell anemia—SCA) or associated with other structural or synthesis variants of hemoglobin, generating a diversity of genotypes corresponding to the SCD group. HbS is a consequence of a single nucleotide substitution at codon 7 of the beta globin gene (*HBB*), which replaces the glutamic acid to the valine amino acid [1]. Under low oxygen concentration HbS can polymerize and cause modifications in the shape and physical properties of erythrocytes, which leads to hemolysis and increased adhesion to erythrocytes, leukocytes, endothelial cells, coagulation factors, platelets and other plasma proteins [2]. The complex molecular interaction with sickled erythrocytes can lead to vaso-occlusive events that are the hallmark of SCD. The vaso-occlusive events occur manly in small vessels, resulting in potentially adverse consequences in different organs [3, 4].

SCD is therefore characterized by complex multisystemic complications that can evolve to chronic organ damage. Pregnancy in SCD women presents increased risk of mortality and morbidity to the mother and to the newborn, particularly due to the aggravated SCD complications in these pregnant women [5]. In recent years, advances in perinatal care, neonatal screening techniques and preventive measures have reduced maternal and perinatal mortality. Nevertheless pregnant women with SCD still have high rates of adverse outcomes compared to the baseline population [6, 7]. Complications in pregnant women with SCD include increased susceptibility to pain crises, sepsis, urinary tract infections, acute chest syndrome, worsening anemia and also increased risk of preeclampsia and eclampsia. Fetal/infants risks include: increased risk of abortion, fetal growth restriction (FGR), stillbirth, small for gestational age newborns and prematurity, possibly related to placental dysfunction owing to the recurrent placental sickle cell events and inflammatory vasculopathy [8, 9].

The placenta is a key organ to guarantee gestational maintenance and appropriate fetal development. Previous studies showed that placentas from pregnant women with SCD have morphological abnormalities, such as increased intravillous fibrin deposit, villous sclerosis, maternal hemoglobin sickling, as well as abnormalities in size, adherence to the uterine wall and localization, suggesting increased risk of uteroplacental insufficiency that could possibly explain the adverse fetal outcomes [10–12]. The morphological SCD placental abnormalities are well reported, however there is lack of information on how the molecular mechanisms, such as epigenetics mechanisms, could affect placental function in SCD pregnancies.

Recent studies have shown that epigenetic mechanisms (heritable control of gene expression not related to DNA sequence), specifically DNA methylation, can be altered in placental tissue in the presence of maternal pathology. These studies revealed that pregnant women with diabetes mellitus and obesity had significant DNA methylation alterations in the placenta, compared to the placenta of healthy pregnant women [13, 14]. Furthermore, a study of placentas from pregnant smokers showed alterations in DNA methylation and these were also associated with decreases in birthweight, suggesting that maternal background may cause epigenetic modifications in the placenta, leading to adverse outcomes [15]. Considering that there are currently no reports on DNA methylation in SCD placentas, this study aimed to evaluate the DNA methylation profile of placentas of pregnant women with SCD (HbSS and HbSC genotypes), compared to uncomplicated controls (HbAA), considering maternal and perinatal outcomes.

## Materials and methods

### Study participants

This study adhered to the tenets of the Declaration of Helsinki and was approved by the Research Ethics Committee of the Faculty of Medical Sciences, University of Campinas under the number 2.502.968. All patients signed an informed consent form prior to sample collection. Pregnant women who comprised the case group were selected from the high-risk outpatient clinic and obstetric room of the University of Campinas (UNICAMP); controls were selected both from the University of Campinas and the Campinas Maternity Hospital. For this case-control study, we included 22 pregnant women with SCD (HbSS = 11 and HbSC = 11) and 21 pregnant women with non-complicated gestation, named control pregnancies (HbAA = 21). Data on maternal and perinatal outcomes were retrieved from careful medical chart review. Diagnosis of HbSS, HbSC and HbAA was performed by clinical and laboratory data, family analysis, Hb electrophoresis and sequencing when necessary at the institution’s clinical laboratory. The control group was composed by uncomplicated pregnant women admitted to childbirth in both maternities and all were at term pregnancy, with elective cesarean, mostly scheduled due to repeat c-section, breech presentation or maternal request. Women with normal delivery, diagnosis of hypertension, diabetes, proteinuria, fetal abnormalities, history of infections, drug use and smoking, were excluded from the control group. Among sickle cell disease cases, route of delivery was defined by the local clinical protocol, according to each case.

### Placental tissue collection

The placental tissue was collected within 3h after childbirth. Previously to sample collection, the placentas were weighed and then washed with phosphate saline solution to withdraw the remaining maternal blood. After cleaning, the placenta was placed on a sterilized surface with the maternal side facing up. The choice of the sampling was based on the insertion of the umbilical cord to ensure representativeness. At the maternal side, four points were chosen for sampling, equidistant from each other, according the protocol guideline [16, 17]. The basal plate layer was removed at the point of the collection to guarantee the villus tissue sampling. After collection, the samples were frozen in liquid nitrogen and stored at -80°C until DNA and RNA extractions. Following the protocol guideline, pictures from maternal and fetal side were taken for each included placenta.

### DNA extraction and bisulfite conversion

Genomic DNA was extracted from approximately 25 mg of placental tissue using the *QIAamp DNA Mini* kit (Qiagen, Hamburg, Germany). DNA purity and quantity were evaluated using NanoDrop (Thermo Fisher, CA, USA) and Qubit (Life Technologies, CA, USA) equipments, respectively. The integrity of DNA samples was assessed through the electrophoresis of 2–5μL of each sample on 1% agarose gel. For bisulfite conversion, 500 ng of DNA were treated using the EZ DNA Methylation kit (Zymo Research, CA, USA) following the manufacturer’s recommendations. All bisulfite-converted DNA samples were stored immediately at -20°C until use.

### DNA methylation analysis

DNA methylation assays were performed using the Illumina Methylation EPIC BeadChip (EPIC) (Illumina, CA, USA). Bisulfite-converted DNA of eight HbSS, eight HbSC and seven control samples were submitted to HM850K hybridization, according to the Illumina Infinium HD methylation protocol (https://support.illumina.com). The EPIC platform assesses the DNA methylation level of 853,307 *loci* around the genome at single-nucleotide resolution. Chips were scanned by Illumina iScan SQ scanner (Illumina, CA, USA) and the fluorescence signals were interpreted with the Bioconductor packages in R environment (v.3.4.4). Annotation of probes was performed from the Illumina files using UCSC version hg19 of the human reference genome. The methylation levels were obtained for each CpG site as beta-values that range from 0 to 1, which is related to the percentage of methylation, from 0 to 100%. Then, the dataset was analyzed using the *minfi* package [18]. For quality control, probes with detection p-value > 0.05 were removed from the dataset. The probes on the EPIC have two different chemistry designs, type I and II, which need to be normalized to make them comparable to each other. This normalization was performed using the *FunNorm* method, which adjusts intensities based on a quantile approach [19]. Adjustments for batch effects were performed using the *ComBat* function implemented in the *ChAMP* package. Probes located on the X and Y chromosomes were removed. In addition, probes associated with known SNPs and those located in no-CpG sites (no-CpG control probes) were also removed from downstream analysis [20], resulting in a total of 735,716 probes for each of the 23 samples (8 HbSS, 8 HbSC and 7 controls).

### Differential methylation analysis

We followed the guidance of Du et al. (2010) and imposed a 0.15 minimum threshold for the difference between mean beta-values of the compared groups. Thus, only probes with absolute difference between groups above 0.15 were taken into account for the downstream analyses. The β-values were log-transformed into M-value, since the M-values present higher homoscedasticity, generating data more homogeneous and less dispersed in the extremes compared to β-values [21]. After that, the comparison analyses between groups were performed using an empirical Bayesian framework linear model from *limma* package [22]. Besides the phenotype, we used gestational age and fetal sex as confounding variables in our models. The fetal sex can contribute to differential methylation in placental tissue; for that we included this variable in our regression analysis to avoid fetal sex interference in the methylation analysis [23]. The Benjamini-Hochberg’s method was applied and the CpGs sites with adjusted p-values (adjP)<0.05 were considered differentially methylated. The analyses were performed comparing the HbSS and HbSC group versus control group independently, thus the differentially methylated position (DMPs), CpG sites, were obtained for both comparisons. SVA package was used, however we did not obtain any significant probes; for that reason we chose not to apply this correction. The *DMRcate* package [24] was also used to identify the differentially methylated regions (DMRs), based on groups of probes that presented the same differentially methylated status (p-value<0.05), where the next consecutive probe was within 1,500 nucleotides. In other words, the *DMRcate* package identify regions that contain CpG sites that present the same methylation status (hyper or hypomethylated) distant up to 1.500 bases between them. DMRs with adjP<0.05 were considered significant. The genes identified in the significant DMRs were submitted to functional enrichment analyses (Biological Processes from Gene Ontology) using WebGestalt, which analyzes against the human reference genome, applying a Benjamini-Hochberg multiple-test adjustment threshold of p<0.05 [25]. To facilitate biological interpretation, the M-values were converted again to β-values.

### DMRs/Genes selection criteria for methylation validation/expression analysis

175 Some DMRs were selected for methylation validation. Initially, as selection criteria, we considered DMRs with greater methylation difference (more than 15%). Subsequently that DMRs were considered according to their gene location, selecting those present in promoter regions and/or 1st exon (regions strongly associated with gene expression regulation). A list of DMRs for each group, HbSS and HbSC, was obtained and their associated genes were identified. Gene ontology analysis, of the DMR-associated genes was performed (Webgestalt - http://www.webgestalt.org/option.php). Finally, we considered genes involved in significant biological processes and/or those associated with placental development and fetus growth such as trophoblastic cell adhesion, migration and proliferation. According to these criteria, four genes were selected from the HbSS group (*PTGFR*, *GPR56*, *GALR2* and *ADCY4*) and three genes from the HbSC group (*SPOCK1*, *THSD7A* and *ADCY4*), with one gene in common between groups (*ADCY4*). These genes are involved in significant biological processes associated with placental development and fetus growth such as trophoblastic cell adhesion, migration, and proliferation.

The DMRs associated to these genes were submitted to bisulfite pyrosequencing for methylation validation (one CpG site present in each DMR, randomly selected, was evaluated). Furthermore, gene expression analysis was performed for each gene selected. The complete table with more information about the selected DMRs is available in (S1 Table).

### Bisulfite pyrosequencing

Pyrosequencing is a quantitative method that measures the DNA methylation levels (in %) for each CpG site in a specific genome region. Thus, this method was used for technical validation of the differentially methylated loci identified by HM850k array. The DNA samples assessed by HM850K were also used in validation experiments with three additional samples per group (total: HbSS = 11; HbSC = 11; control group = 10).

For each DMR, one CpG site was evaluated for methylation level, where four CpGs sites were analyzed in the HbSS group: cg03949391-*PTGFR*, cg03989617-*GPR56*, cg07274618-*GALR2* and cg23179456- *ADCY4*; and 3 CpGs sites were analyzed for the HbSC group: cg24847829-*SPOCK1*, cg24676244-*THSD7A* and cg23179456-*ADCY4*. Firstly, PCR was performed using 50 ng of bisulfite-converted DNA in a final volume of 50 μL, using specific primers for each CpG site (Table 1), which were constructed by the software PiroMark (Qiagen, Hamburg, Germany). Next, 40 μL of the amplified product was used for pyrosequencing using PyroMark Gold Q96 Reagents kit and the PyroMark Q96 pyrosequencer (Qiagen, Hamburg, Germany), following the manufacturer’s recommendations. Pyrosequencing was performed for each region in duplicate and the mean of these duplicates was calculated and used for case and control group comparison. Furthermore, correlation analysis was performed between array and pyrosequencing data using Pearson method in normal data and Spearman method in no-normal data.

**Table 1:** Primers sequences used for pyrosequencing analysis.

CpG (Probe ID)	Gene	Primers sequences (5’- 3’)	Annealing (°C)	Length (bp)
cg03949391	*PTGFR*	F: TAGTTAGGTGTAGAGGGATTTTAGGA	56	250
		R:[btn]CAACCTCTAAAAAAATAATACCTTATCAT		
		Seq: GGTGGAATTTGAGGTAG	-	
cg03989617	*GPR56*	F: [btn] AGGAGAGGGGTGTTTTTTTATTAA	58	249
		R: CACCTACTATCCAACCCTTATT		
		Seq: CAAAACAAAACAAACAACTAAT	-	
cg07274618	*GALR2*	F: TAGGGGTTTTTTTTTGAGGGTATTTT	60	408
		R: [btn]AAAAAAACCTTACCTCATCTAAAAC		
		Seq: GGAAGTAGGTATAAG	-	
cg24847829	*SPOCK1*	F: [btn]AGTTATTGGTTATTGTTTAGGAAATT	56	620
		R: CAAAAAAAACCTTTCCCTTAACTAT		
		Seq: AATCCCCTATAATTAAAC	-	
cg24676244	*THSD7A*	F: AAGGAGTAGAGGGGGTTGG	60	359
		R: [btn]CTCAAATACTACTCCCCACACAA		
		Seq: GGAGAGGGGTTAGTT	-	
cg23179456	*ADCY4*	F: [btn]AGTAGATTTAGAAGGGTAGAGTG	59	624
		R: CCAAATCCTACCCTCCTAAC		
		Seq: ACCCTAACCAACC	-	

F: forward sequence, R: reverse sequence, Seq: sequence used in the pyrosequencing reaction, btn: sequence with biotin marking, bp: base pairs.

### Gene expression analysis

#### RNA extraction and cDNA synthesis

From the DMRs assessed by bisulfite pyrosequencing, the associated genes were identified and submitted to gene expression evaluation. This analysis was performed for the HbSS group (n = 11), HbSC group (n = 11) and control group (with 11 additional samples, n = 21). The total RNA from the placental tissue (chorionic villous) was extracted using TRIzol Reagent (Life Technologies, MD, EUA), and RNeasy Mini Kit (Qiagen, Hamburg, Germany) following the manufacturer’s protocol. The concentration and purity of the total RNA were assessed using the NanoDrop 2000 spectrophotometer (Thermo Scientific, CA, EUA). RNA samples were stored at -80°C until use. Subsequently, about 1 μg of RNA was treated with DNAse I (Life Technologies, CA, USA) and then cDNA was synthesized using the RevertAid First Strand cDNA Synthesis Kit (Thermo Scientific, MA, USA), according to the manufacturer’s instructions. The cDNA was stored at -20°C until use.

#### Real time PCR

Quantitative PCR was performed in a 12 μl reaction final volume containing 3 μl of cDNA (10 ng), 6 μl SYBR Green PCR Master Mix (Applied Biosystems, CA, USA) and 6 μl of specific primers. The summary of the primer sequences used for the qPCR are shown in Table 2. The qPCR reaction was carried out in duplicate using ABI StepOnePlus Real Time PCR (Applied Biosystem, CA, USA) and the cycling steps included: 95°C for 10 min, 95°C for 15 s (40 cycles) and 60°C for 1 min (40 cycles). The standard equation (2^(-ΔCt)) was used to calculate the relative changes in gene expression, as reported by Livak [26]. The data were normalized by the reference genes *ACTB* and *GAPDH* and then converted to fold changes.

**Table 2:** Primer sequences used for real time PCR analysis.

Gene	Primers sequence (5’-3’)	Length (bp)	Primer concentration
*PTGFR*	F: GATGACAAGATGTCTGGACTGC	118	150 nM
	R: CAGGAGACACTAGCTGTTTGGA		
*GPR56*	F: TGCTGATGGTCTCCTCGGTG	108	70 nM
	R: CAATGGTGACAAGGCAGGCC		
*GALR2*	F: GCACTTCCTCATCTTCCTCACC	73	150 nM
	R: CAGATACCTGTCCAGGGAGACG		
*SPOCK1*	F: TGCTGTGAGCTGTGAAGAGGAG	113	70 nM
	R: CTTTGTCCTTTGGTCCCAGCTC		
*THSD7A*	F: TCATGTTATGATGGACAGTGCTAT	100	150 nM
	R: CATTTATACCATCTGACCTTTGAC		
*ADCY4*	F: CCCAACATCATCAGACTGCCCT	120	100 nM
	R: CAGCAGTGCATGGAGTATGGGA		
*BAC*	F: TGACCCAGATCATGTTTGAGACC	81	150 nM
	R: CAGAGGCGTACAGGGATAGCA		
*GAPDH*	F: AAGATCATCAGCAATGCCTCCT	96	150 nM
	R: GGTCATGAGTCCTTCCACGATAC		

F: forward sequence, R: reverse sequence, bp: base pairs, nM: nano Molar.

### Statistical analysis

Statistical analysis for bisulfite pyrosequencing and gene expression data were performed using GraphPad Prism version 5.0 software (GraphPad Software, CA, USA). The Mann-Whitney *U* test was used for non-normal distribution data and the Student’s unpaired *t* test was used for normal distribution data. The statistical methods were applied for the case group compared to the control group (HbSS versus HbAA and HbSC versus HbAA) and *p* value <0.05 was considered to be statistically significant. Linear correlation was used to analyze the correlation between DNA methylation at specific CpG sites and gene expression data. For this analysis, the normal data was submitted to Pearson method and the out-normality data was submitted to Spearman method.

## Results

### Clinical characteristics

Clinical characteristics of mothers and offspring are shown in Table 3. This study included 11 pregnant women with HbSS, 11 pregnant women with HbSC and 21 healthy pregnant women without complications during pregnancy (HbAA—control group). The HbSS and HbSC genotypes were compared separately with the HbAA genotype. The comparison between case and control groups showed clinical characteristics (gestational age, body mass index, birth height, birth weight and placental weight) statistically different for HbSS and HbSC groups. Among the groups analyzed, pregnant women with HbSS had lower gestational age at delivery (36.44 ± 1.77 weeks, p<0.0001), lower body mass index prior to pregnancy (22.3 ± 3.1, p = 0.0125), lower birth height (44.95 ± 3.67 grams, p = 0.0005), lower birth weight (2397 ± 457.85 grams, p<0.0001) and lower placental weight (425.27 ± 88.38 grams, p<0.0001). Furthermore, the HbSS group presented higher frequency of sickle-related complications during pregnancy than the HbSC group, which included: vaso-occlusive crises (81.8 vs. 63.6%), acute chest syndrome (18.2 vs. 9.1%), prematurity (54.5 vs. 36.4%), FGR (18.2 vs. 0%), perinatal mortality (9.1 vs. 0%) and infection during pregnancy (54.5 vs. 18.2%). In order to reduce adverse maternal and fetal outcomes, programmed blood transfusion is a protocol procedure in our institution at 28 weeks [27]. Thus, prophylactic blood transfusions were performed in 10 pregnant women with HbSS and in 8 pregnant women with HbSC. Four women (HbSS: 1 and HbSC 3) did not receive prophylactic transfusions for different reasons. In the HbSS patient, preterm birth occurred at 28 weeks, before the beginning of the scheduled transfusion, and neonatal death occurred shortly after childbirth. The other three HbSC patients had difficulty to adhere to the transfusion treatment, due to personal issues.

**Table 3:** Clinical characteristics, maternal and perinatal outcomes among cases (HbSS and HbSC) and controls.

Characteristics	HbSS group n (%) ^a^	HbSC group n (%) ^a^	Control group n (%)
**Total pregnancies (n)**	11	11	21
**Maternal age (years) mean ± SD**	26.81 ± 4.56	25.27 ± 7.02	28.57 ± 3.49
**Pre-pregnancy BMI (kg/m** ^**2**^ **)**	22.3 ± 3.1* ^b^	22.4 ± 2.0** ^b^	26.5 ± 3.1
**Maternal weight gain during gestation (kilogram)**	7.7 ± 3.3	8.5 ± 2.1	12.3 ± 5
**Nulliparous (n)**	6 (54.5)	7 (63.6)	6 (29.6)
**Gestational age at birth (weeks) mean ± SD**	36.44 ± 1.77*** ^b^	36.68 ± 3.94*** ^b^	39.82 ± 0.74
Preterm birth (< 37 weeks)	5 (54.5)	4 (36.4)	-
Preterm birth (< 34 weeks)	1 (9.1)	1 (9.1)	-
**Route of delivery (n)**			
Cesarean section	11 (100)	11 (100)	21 (100)
Labor ^d^	3 (27.3)	4 (36.4)	-
**Pre-eclampsia**	0	1 (9.1)	-
**Birth height (cm) mean ± SD**	44.95 ± 3.67*** ^b^	47.66 ± 5.37** ^b^	49.66 ± 1.25
**Birthweight (grams) mean ± SD**	2397 ± 457.85*** ^b^	2952 ± 484.37** ^b^	3636 ± 327.30
FGR	2 (18.2)	0	-
**Placental weight (grams) mean ± SD**	425.27 ± 88.38*** ^c^	513.63 ± 96.22*** ^c^	745.14 ± 114.15
**Newborn sex (n)**			
Male	7 (63.6)	7 (63.6)	11 (52.4)
Female	4 (36.4)	4 (36.4)	10 (47.6)
**Sickle-related complications during pregnancy (n)**			
Vaso-occlusive crises	9 (81.8)	7 (63.6)	-
Acute chest syndrome	2 (18.2)	1 (9.1)	-
Infection during pregnancy	5 (54.5)	2 (18.2)	-
Programmed blood transfusion	10 (90.9)	8 (72.7)	-
Hospital admission during pregnancy	8 (72.7)	5 (54.5)	-
Perinatal mortality	1 (9.1)	0	-

Pre-pregnancy BMI: body mass index prior to pregnancy, FGR: fetal growth restriction, SD: standard deviations

^a^: compared with the control group

^b^: Mann-Whitney test U

^c^: Unpaired t test

**p<0.01

*** p<0.001.

^d^: Women that presented a period of labor or that started with a spontaneous labor before the need for a c-section.

### Placental gross evaluation

**Fig 1:**
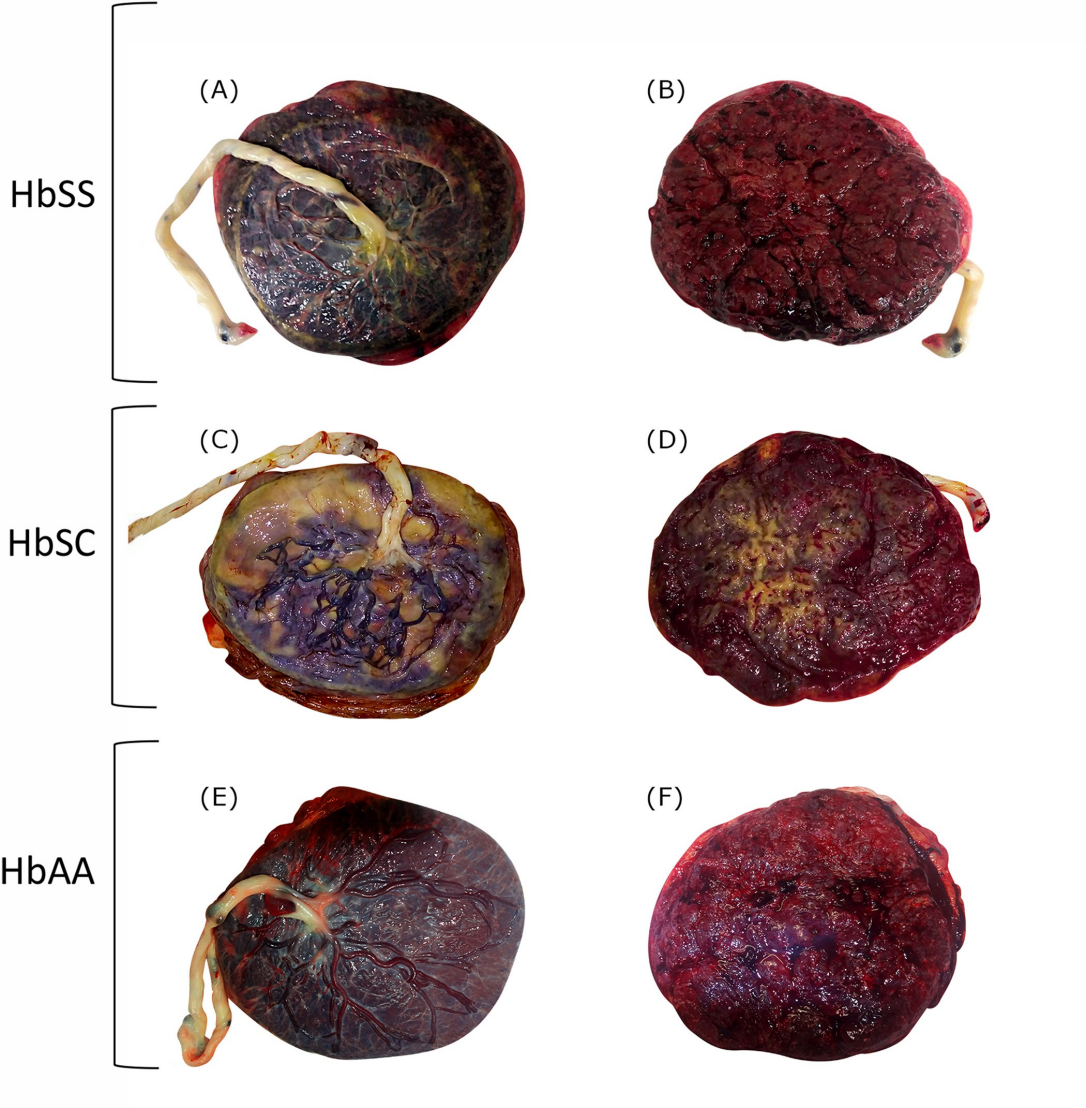
Gross evaluation of one selected placenta from each of the three groups of patients considered: HbSS, HbSC and HbAA (without SCD). Fetal (A) and maternal (B) sides of the placenta from a HbSS patient with a term cesarean delivery (37 week gestation) with multiple hospital admissions for worsening anemia and previous exchange transfusion at 28 weeks. Fetal (C) and maternal (D) sides of the placenta from a HbSC patient, also delivered by cesarean at term, due to maternal request (37 weeks) with programmed blood transfusions during third trimester and no severe complications. Fetal (E) and maternal (F) sides of the placenta from a patient without SCD, delivered at 39 weeks, by Cesarean section due to 2 previous cesareans. In the HbSS and HbSC placentas it is possible to observe increased subchorionic fibrin deposition and calcifications (non-specific alterations). For methylation analysis, villous tissue was sampled.

For all placentas considered, pictures of the maternal and fetal sides were obtained, and also placental weight and volume. As an example of the gross examination of placentas from the three groups of patients: HbSS, HbSC and Control (without SCD), is presented, to demonstrate abnormal findings (Fig 1). These pictures of placentas are not representative of all included cases. Images are examples for each considered group. Note increased subchorionic fibrin deposition in the HbSC placenta, in plaques around 50% of the surface. (Fig 1C and 1D). In the HbSS it is also increased from the control. The HbSC shows more gritty areas, related to calcifications. (Fig 1A and 1B).

### Methylation analysis at CpG sites–DMPs

**Fig 2:**
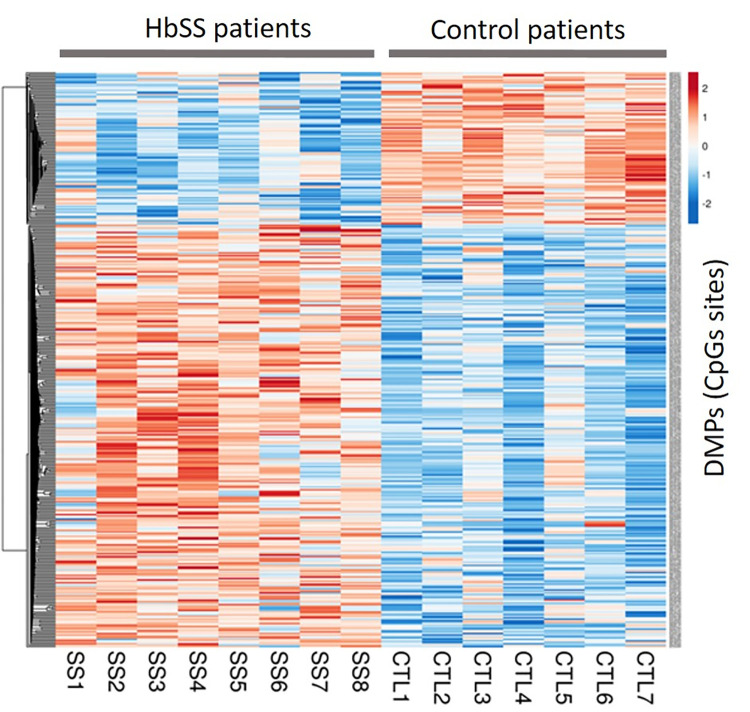
Heatmap generated from the 396 DMPs obtained in the HbSS group compared to the control group. The rows represent each DMP and the columns each patient in the HbSS group and controls (CTL). The colors represent the methylation levels; the more red the more methylated and the more blue the less methylated. From these 396 DMPs, a total of 68 DMPs were in common with those obtained in the HbSC group.

Comparative analysis of genome-wide CpG methylation levels in placentas from case and control groups showed more proportion of hypermethylated DMPs in HbSS and HbSC groups. In the comparison between HbSS versus control group were identified 396 DMPs, among these, 291 DMPs (73.5%) were hypermethylated and 105 DMPs (26.5%) were hypomethylated. For better visualization of this hypermethylation status in the HbSS group, a heatmap was created from the methylation value of each DMPs obtained from the comparison between the HbSS group versus the control group (Fig 2).

**Fig 3:**
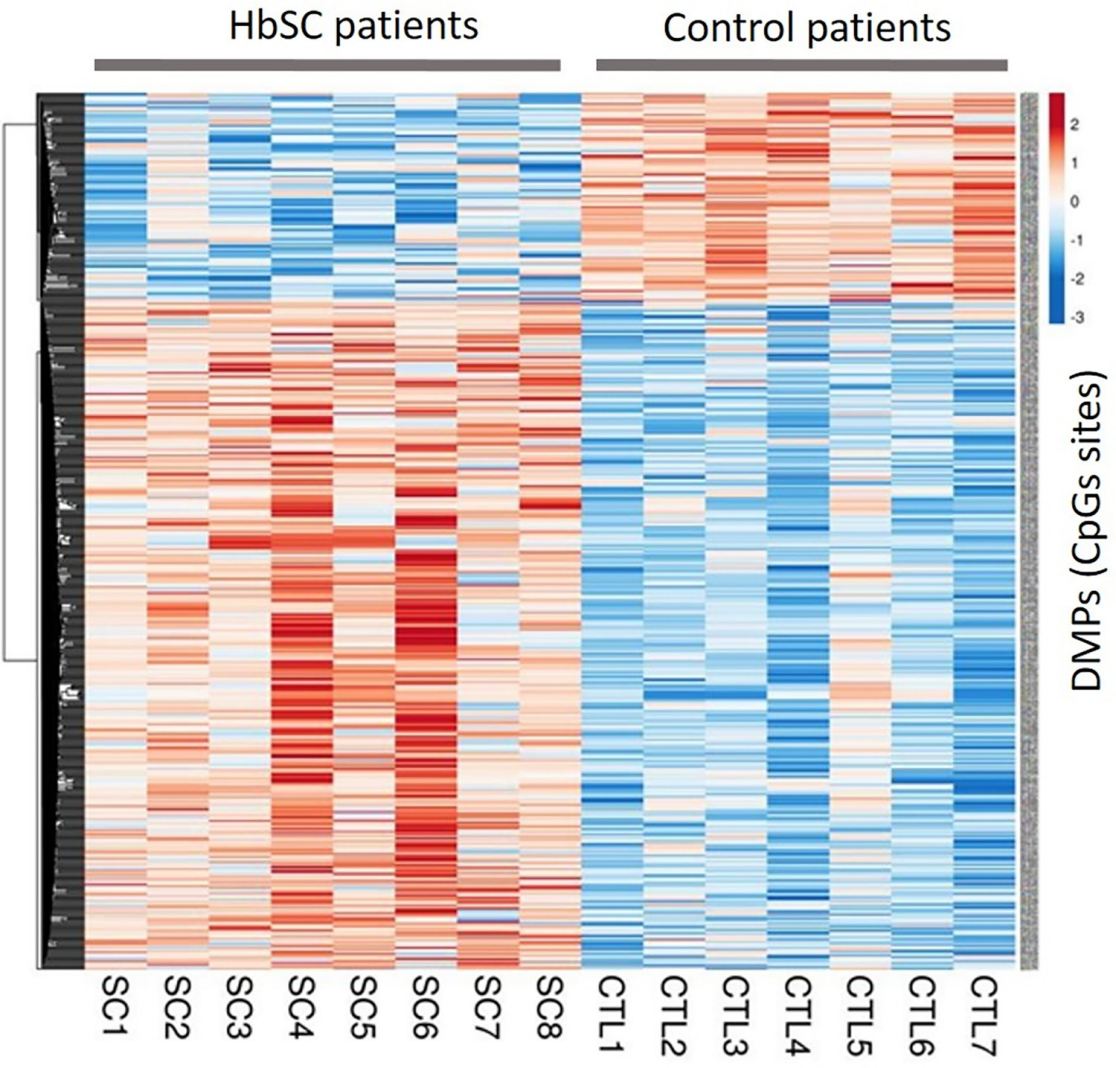
Heatmap generated from the 581 DMPs obtained in the HbSC group compared to the control group. The rows represent each DMP and the columns each patient in the HbSC group and controls (CTL). The colors represent the methylation levels; the more red the more methylated and the more blue the less methylated. From these 581 DMPs, a total of 68 DMPs were in common with those obtained in the HbSS group.

In the comparison between HbSC and control groups 581 DMPs were identified, of which 443 DMPs (76.2%) were hypermethylated and 138 DMPs (23.8%) were hypomethylated. A hypermethylation status is also present in the HbSC group which could be visualized from the heatmap in Fig 3. A total of 68 hyper and 6 hypomethylated DMPs were in common for both genotypes groups.

The lists of DMPs used to enable heatmap construction, with comparisons: HbSS vs Control and HbSC vs Control, are included in (S2 Table).

**Fig 4:**
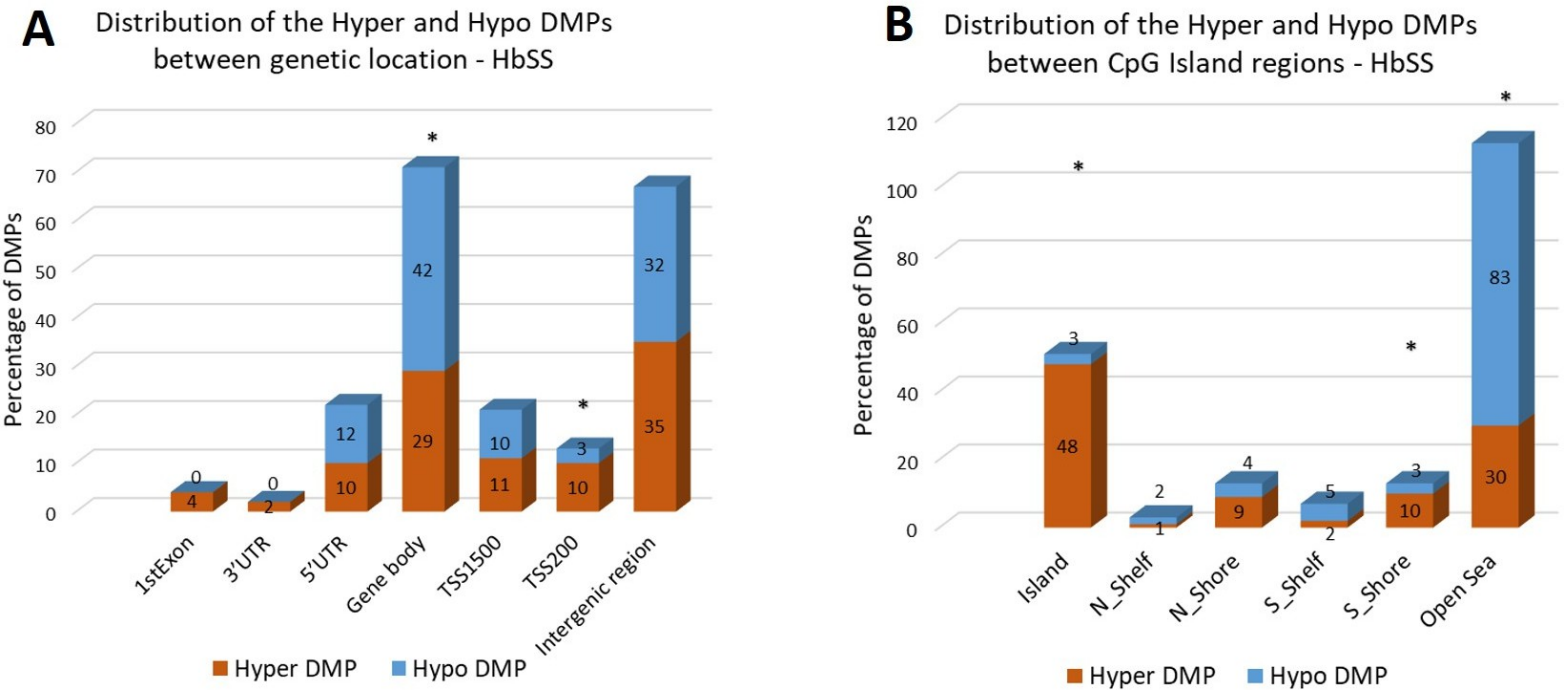
(**A**) The distribution of hyper DMPs and hypo DMPs according to their distance from the promoter. *TSS1500*, 200 to 1500 base pairs upstream of the transcription start site (TSS); *TSS200*, 200 base pairs upstream of the TSS; *5′UTR*, 5′ untranslated region; *1st Exon*; *3′UTR*, 3′ untranslated region. **(B)** The distribution of hyper DMPs and hypo DMPs in different genomic region types. *Island*, a CpG site located within a CpG island; *Shore*, a CpG site located < 2 kilobases from a CpG island (N_: located at North; S_: located at South); *Shelf*, a CpG site located > 2 kilobases from a CpG island; *Open sea*, a CpG site not in an island or annotated gene. Data of DMPs obtained from the comparison between HbSS vs Control groups. *: group of DMPs (hyper or hypomethylated) statistically more frequent in a specific region (p<0.05; chi-square distribution test).

Distribution analysis of DMPs in the genomic region types was performed and the data are presented in Figs 4 (HbSS group) and 5 (HbSC group). The DMPs distribution among the genetic and CpG island regions is shown, as well as the frequency of hyper and hypomethylated DMPs in each region.

**Fig 5:**
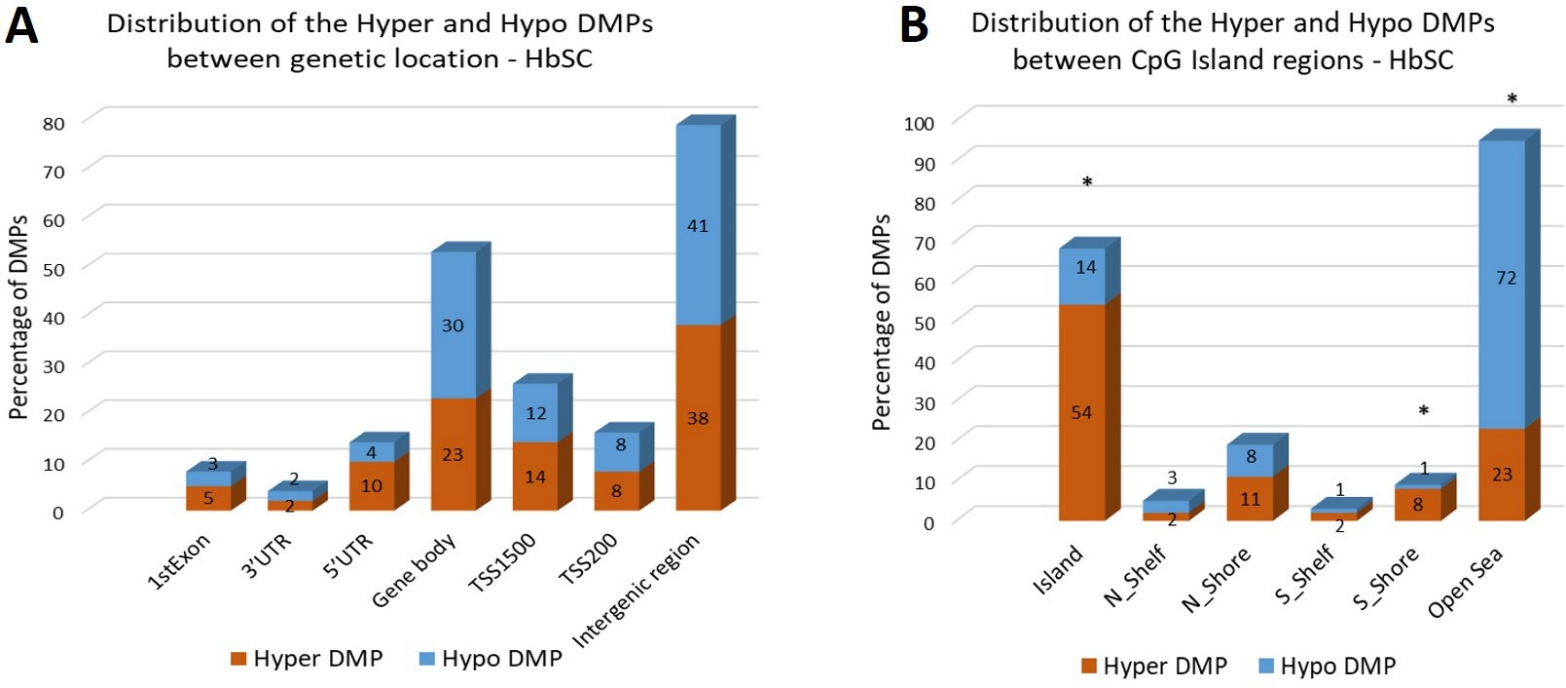
(**A**) The distribution of hyper DMPs and hypo DMPs according to their distance from the promoter. *TSS1500*, 200 to 1500 base pairs upstream of the transcription start site (TSS); *TSS200*, 200 base pairs upstream of the TSS; *5′UTR*, 5′ untranslated region; *1st Exon*; *3′UTR*, 3′ untranslated region. **(B)** The distribution of hyper DMPs and hypo DMPs in different genomic region types. *Island*, a CpG site located within a CpG island; *Shore*, a CpG site located < 2 kilobases from a CpG island (N_: located at North; S_: located at South); *Shelf*, a CpG site located > 2 kilobases from a CpG island; *Open sea*, a CpG site not in an island or annotated gene. Data of DMPs obtained from the comparison between HbSC vs Control groups. *: group of DMPs (hyper or hypomethylated) statistically more frequent in a specific region (p<0.05; chi-square distribution test).

For the HbSS group, the distribution of DMPs in genetic location showed hyper DMPs most abundant in intergenic region (35%), whereas hypo DMPs were mainly distributed in gene body (42%). We also determined the enrichment of the DMPs by calculating the ratio of hyper DMPs to hypo DMPs in each region. The results indicated that hyper DMPs were enriched in TSS200 (p = 0.0373), whereas hypo DMPs were mainly distributed in gene body (p = 0.0239) (Fig 4A). The distribution of DMPs in CpG Island region was also performed and showed greater frequency of hyper DMPs in CpG Island (48%), and greater frequency of hypo DMPs in Open Sea region (83%). We also determined the enrichment of the DMPs and observed that hyper DMPs were enriched in CpG island (p<0.0001), as well as in S_Shore regions (p = 0.0306), and hypo DMPs were enriched in Open Sea region (p<0.0001) (Fig 4B).

The distribution of DMPs in genetic location was also performed in the HbSC group and the results showed both hyper DMPs (38%) and hypo DMPs (41%), most abundantly distributed in intergenic regions. The enrichment analysis by calculating the ratio of hyper DMPs to hypo DMPs in each region was performed. The enrichment analysis by calculating the ratio of hyper DMPs to hypo DMPs in each region was performed; however, we did not observe any region enriched with hyper or hypo DMPs (Fig 5A). The DMPs in CpG Island region were submitted to distribution analysis and the results showed hyper DMPs most abundant in CpG Island (54%), whereas hypo DMPs more distributed in Open Sea region (72%). After enrichment analysis for DMPs for each CpG Island region we observed that hyper DMPs were enriched in CpG island (p<0.0001), as well as S_Shore regions (p = 0.0101), and hypo DMPs were enriched in Open Sea region (p<0.0001) (Fig 5B).

### Differentially Methylation Regions analysis—DMRs

For the HbSS group, our regional analysis identified 57 DMRs which showed statistically significant difference (adjusted p-value <0.05), of which 51 DMRs (89.5%) were hypermethylated and 6 DMRs (10.5%) were hypomethylated. Most of them were located in the promoter/1^st^ exon (43.8%) and in intronic regions (28%). The methylation level (Δβ) ranged in 0.31 (the highest hypermethylated DMR) to -0.23 (the lowest hypomethylated DMR) (S3 Table). The comparison between HbSC and control groups revealed 106 DMRs, including 91 DMRs hypermethylated (85.8%) and 15 hypomethylated (14.2%), being more frequent in the promoter/1^st^ exon (53.7%) and intergenic regions (21.7%), with methylation levels ranging from 0.25 to -0.33 the. The lists of DMRs obtained from HbSS and HbSC groups are available in (S3 Table).

Among all the statistically significant DMRs, 50 genes were identified for the HbSS group and 87 genes for the HbSC group. The analysis of enrichment pathways was performed for both gene groups and among the statistically significant biological processes (FDR <0.05). We highlighted the most significant biological processes for the HbSS group: mesenchyme development, pattern specification process and the adenylate cyclase-modulating G protein-coupled receptor signaling pathway. For the HbSC, the most significant biological processes were animal organ morphogenesis, tissue development, circulatory system development and the central nervous system neuron differentiation (Table 4).

**Table 4:** The GO terms for differentially methylated genes between cases (HbSS and HbSC) and controls groups. *GALR2*, *PTGFR*, *ADCY4*.

Group	GO No.	GO term	Genes	FDR
HbSS	GO: 0007188	adenylate cyclase-modulating G protein-coupled receptor signaling pathway	*ADCY4*, *CASR*, *GALR2*, *LHCGR*, *PTGFR*, *SSTR4*	0.035863
	GO:0007189	adenylate cyclase-activating G protein-coupled receptor signaling pathway	*ADCY4*, *GALR2*, *LHCGR*, *PTGFR*, *SSTR4*	0.035863
	GO:0007187	G protein-coupled receptor signaling pathway, coupled to cyclic nucleotide second messenger	*ADCY4*, *CASR*, *GALR2*, *LHCGR*, *PTGFR*, *SSTR4*	0.035863
	GO:0060485	mesenchyme development	*BNC2*, *GBX2*, *GSC*, *ROBO2*, *SIX1*, *TGFB1|1*	0.035863
	GO:0001708	cell fate specification	*GSC*, *LBX1*, *SIX*, *SOX1*	0.041317
	GO:0007389	pattern specification process	*GBX2*, *GSC*, *LBX1*, *ROBO2*, *SIX1*, *ZIC1*	0.041317
	GO:0019933	cAMP-mediated signaling	*ADCY4*, *GALR2*, *LHCGR*, *PTGFR*, *SSTR4*	0.041628
	GO:0021884	forebrain neuron development	*GBX2*, *ROBO2*, *SOX1*	0.041628
	GO:0048665	neuron fate specification	*LBX1*, *SIX1*, *SOX1*	0.049047
HbSC	GO: 0021953	central nervous system neuron differentiation	*EPHA4*, *GABRB1*, *GBX2*, *MNX1*, *NFIB*, *NR2E1*	0.0017779
	GO: 0021954	central nervous system neuron development	*EPHA4*, *GABRB1*, *GBX2*, *NFIB*, *NRAE1*, *ROBO2*	0.0017779
	GO: 0009887	animal organ morphogenesis	*AJAP1*, *GBX2*, *GATA4*, *HAND1*, *HOXD11*, *LFT*, *LHX9*, *NKX3-2*, *NFIB*, *OLFM1*	0.0045345
	GO: 0035295	tube development	*EPHA4*, *GBX2*, *GATA4*, *HAND1*, *HOXD11*, *LEPR*, *HLA-G*, *NKX3-2*, *NFIB*, *NR2E1*	0.0045345
	GO: 0035239	tube morphogenesis	*EPHA4*, *GBX2*, *GATA4*, *HAND1*, *HOXD11*, *LEPR*, *HLA-G*, *NFIB*, *NR2E1*, *PRKD2*	0.0099342
	GO: 0001822	kidney development	*EPHA4*, *HOXD11*, *KCNJ8*, *NPHS2*, *ROBO2*, *SIX1*, *TP73*	0.010114
	GO: 0009888	tissue development	*AJAP1*, *BARHL2*, *EPHA4*, *EVC*, *GBX2*, *HAND1*, *HOXD11*, *LTF*, *LGR6*	0.011194
	GO: 0072001	renal system development	*EPHA4*, *HOXD11*, *KCNJ8*, *ROBO2 SIX1*, *TP73*, *WT1*	0.011194
	GO: 0072073	kidney epithelium development	*EPHA4*, *HOXD11*, *NPHS2*, *ROBO2 SIX1*, *WT1*	0.012108
	GO: 0072359	circulatory system development	*GBX2*, *GATA4*, *HAND1*, *LEPR*, *HLA-G*, *NR2E1*, *OLFM1*, *KCNJ8*, *PRKD2*, *ROBO2*	0.013806

GO: Gene ontology.

### Methylation validation of the DMRs

**Fig 6:**
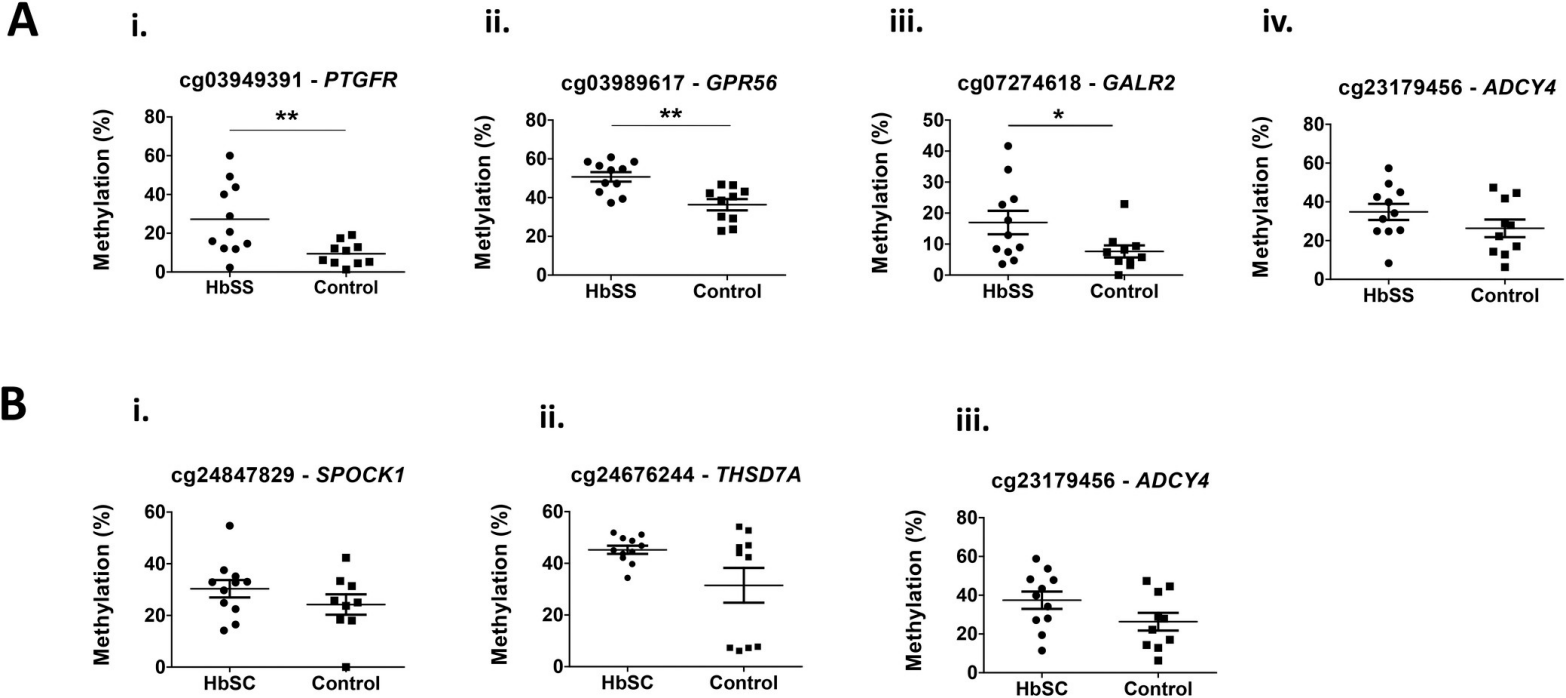
Methylation data from pyrosequencing analysis in the HbSS and HbSC groups compared with the control group (CON). A: CpGs sites analyzed in the HbSS group. i cg03949391-PTGFR; ii cg3989617-GPR56; iii cg0727418-GALR2 and iv cg23179456-ADCY4. B: CpGs sites analyzed in the HbSC group. i cg24847829-SPOCK1; ii cg24676244-THSD7A and iii cg23179456-ADCY. *p<0.05, **p<0.01 (Student’s unpaired t test).

To confirm the array data, bisulfite pyrosequencing was used to measure the methylation level in the selected DMRs (four DMRs for the HbSS group and three DMRs for the HbSC group). The analyses were performed at one CpGs sites for each DMR. The assessed CpGs included: cg03949391-*PTGFR*, cg03989617-*GPR56*, cg07274618-*GALR2* and cg23179456-*ADCY4* for the HbSS group and cg24847829-*SPOCK1*, cg24676244-*THSD7A* and cg23179456-*ADCY4* for the HbSC group. The methylation level was performed using the same samples initially analyzed with three additional samples per group (HbSS = 11, HbSC = 11, control group = 10). The methylation analyses between case and control groups were validated for three of the four assessed CpGs sites in the HbSS group (cg03949391-*PTGFR*, cg03989617-*GPR56* and cg07274618- *GALR2)*. No CpG site achieved statistically significant difference for the HbSC group; p-values <0.05 were considered statistically significant (Fig 6).

Correlation analyses between array and pyrosequencing data were performed and a significant correlation (p<0.05) was reached for all analyzed CpGs in both HbSS and HbSC groups (data presented in Table 5).

**Table 5:** Correlation analyses for array and pyrosequencing methylation data.

Group	CpG site-Gene	r^2^	p-value
HbSS	cg03949391-*PTGFR* ^a^	0.952	<0.0001
	cg03989617-*GPR56* ^b^	0.9565	<0.0001
	cg07274618- *GALR2* ^a^	0.9274	<0.0001
	cg23179456- *ADCY4* ^b^	0.6822	0.0051
HbSC	cg24847829- *SPOCK1* ^b^	0.706	0.0048
	cg24676244- *THSD7A* ^a^	0.7882	0.0005
	cg23179456- *ADCY4* ^b^	0.8451	<0.0001

^a^ Spearman method

^b^ Pearson method.

### Expression analysis of differentially methylated genes

**Fig 7:**
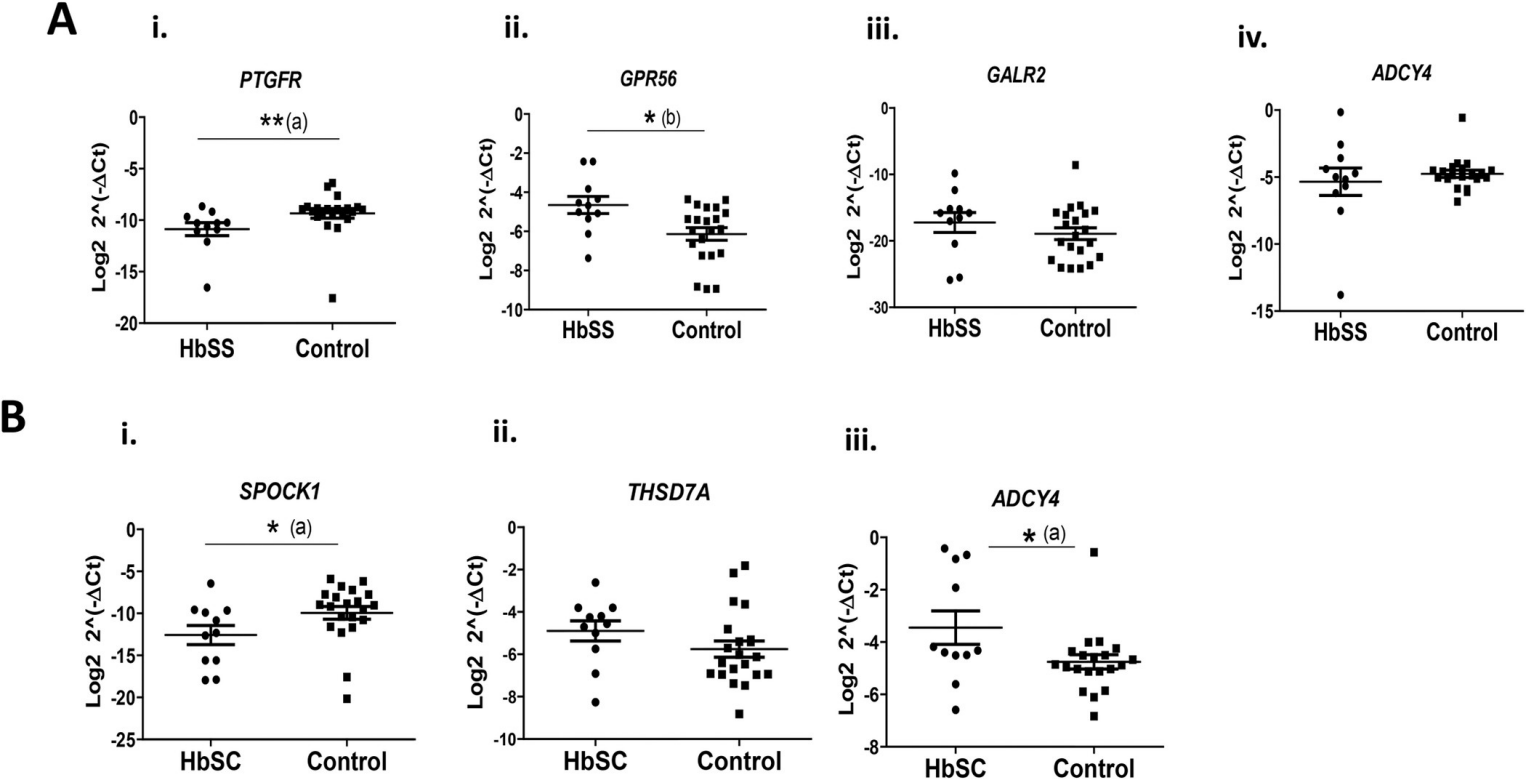
Expression levels of genes in the HbSS and HbSC groups compared with the control group (CON). **A**: Genes assessed in the HbSS group. i *PTGFR*; ii *GPR56*; iii *GALR2* and iv *ADCY4*. **B**: Genes evaluated in the HbSC group. i *SPOCK1*; ii *THSD7A* and iii *ADCY4*. *p<0.05, **p<0.01, (a) Mann-Whitney *U* test, (b) Student’s unpaired *t* test.

In order to evaluate the expression levels of the genes at selected DMRs, quantitative PCR was performed for *PTGFR*, *GPR56*, *GALR2* and *ADCY4* genes (HbSS group), and for *SPOCK1*, *THSD7A* and *ADCY4* genes (HbSC group). The expression levels of all these genes (n = 6) were analyzed in relation to the control group. The comparison between the HbSS group and the control group showed the *PTGFR* gene downregulated (-2.97-fold, p = 0.0062) and the *GPR56* gene upregulated (3.0-fold, p = 0.0103), with no expression difference obtained for *GALR2* (-1.03-fold, p = 0.306) and *ADCY4* (-1.03-fold, p = 0.725) genes. For the HbSC group, the analyses revealed the *SPOCK1* gene downregulated (-2.40-fold, p = 0.0263), the *ADCY4* gene upregulated (1.80-fold, p = 0.0499) and did not find statistical difference for the *THSD7A* gene (1.16-fold, p = 0.1846), when compared to the control group (Fig 7). The data of gene expression levels for each studied cohort are available in (S4 Table).

### Correlation analysis between methylation and expression data

**Fig 8:**
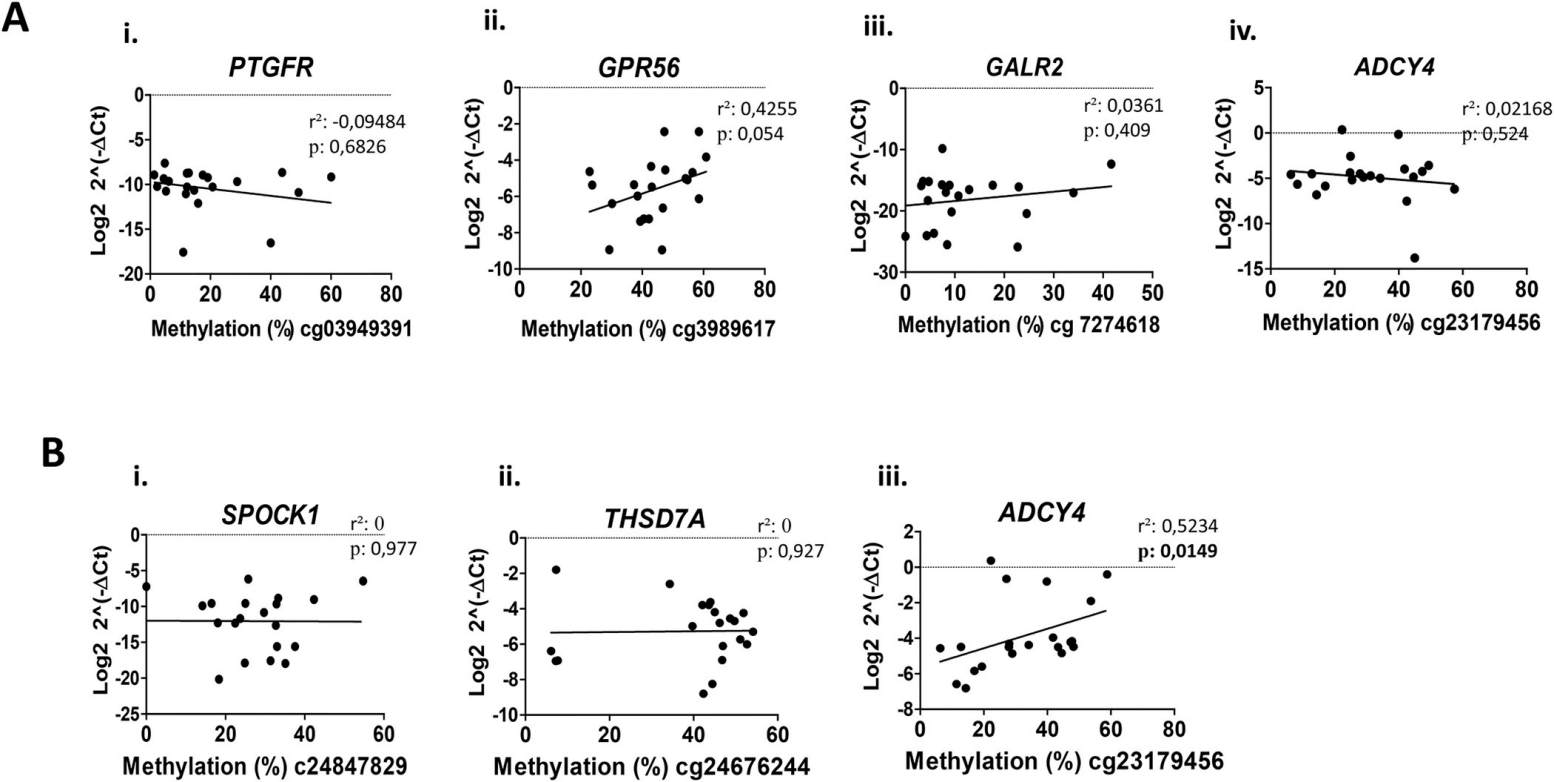
Correlation analyses between methylation and expression data performed in genes from case and control groups. **A**: Analysis in the HbSS group. i *PTGFR*; ii *GPR56*; iii *GALR2* and iv *ADCY4*. **B**: Analysis in the HbSC group. i *SPOCK1*; ii *THSD7A* and iii *ADCY4*. (a) Spearman method. p-values <0.05 are indicated in bold.

The F tests between methylation and expression data was applied and the results for the HbSS group revealed just a trend to positive significant correlation for the *GPR56* (r = 0.42, p = 0.054) gene, and no significant correlation for *PTGFR* (r = 0.09, p = 0.682), *GALR2* (r = 0.03, p = 0.409) and *ADCY4* (r = 0.02, p = 0.524) genes. The tests for the HbSC showed no significant correlation for *THSD7A* (r = 0, p = 0.927) and *SPOCK1* (r = 0, p = 0.977) genes, and interestingly it presented a positive correlation for the *ADCY4* gene (r = 0.52, p = 0.0149) (Fig 8).

In summary our results revealed a hypermethylation status of DMPs and DMRs in both groups, HbSS and HbSC, when compared with the control group. Additionally, three from the four genes selected in the HbSS group were validated through pyrosequencing (*PTGFR*, *GPR56* and *GALR2*), while none of the three genes selected in the HbSC group reached validation. The expression gene analysis showed that *PTGFR* (downregulated) and *GPR56* (upregulated) are differentially expressed in placentas of women with sickle cell anemia as well as *SPOCK1* (downregulated) and *ADCY4* (upregulated) in placentas of women with hemoglobinopathy HbSC. Furthermore, the results of the correlation between methylation and expression data revealed a trend to positive correlation for the *GPR56* gene (HbSS group) and positive correlation for the *ADCY4* gene (HbSC group).

## Discussion

Pregnant women with SCD require a high-risk multidisciplinary antenatal care due to increased risk of maternal and perinatal complications such as acute chest syndrome, pre-eclampsia, infection, spontaneous abortion, preterm birth and FGR. The placenta of women with SCD can present several morphological abnormalities, which may favor maternal and fetal complications. The placental abnormalities have been well described and reported in SCD, however little is known about the molecular mechanisms involved, including gene expression and regulation in this tissue. One of the few studies about gene expression in placentas from this group of patients showed altered gene expression in the inflammatory pathway, indicating placental molecular alteration related to SCD [28]. DNA methylation is a gene regulation mechanism, and it has been intensely studied in human placenta from complicated pregnancies [13, 14, 29]. To our knowledge, so far there is no study assessing DNA methylation in placenta from women with SCD, being this the aim of the study.

In our results, the HbSS and HbSC groups showed a higher frequency of hypermethylated DMPs, presenting a rate of 73.5% and 76.2%, respectively, when compared with the control group. These findings suggest that the presence of HbSS and HbSC can alter placental DNA methylation in a similar way (hypermethylation status), what could be explained by the common pathophysiology of these SCD. However, a small number of common hypermethylated DMPs was shared between both genotypes, indicating an intra-specific genotype effect for methylation at particular CpG sites. A previous study has reported that DNA can suffer hypermethylation under in vitro hypoxic conditions (24h of 1% oxygen) in cultured human placental trophoblasts, which was also associated with non-differentiation of villous cytotrophoblast to syncytiotrophoblast [30]. Pregnant women with SCD can have decreased oxygen levels in the blood circulation due to many factors such as: the presence of sickled erythrocytes, which are less capable of transporting oxygen; vaso-occlusion events, which worsen the maternal oxygen conditions; and the underlying physiological changes of pregnancy, which can compromise the maternal oxygen reserves [31]. All these events can favor decreased placental oxygen circulation, leading to a hypoxic environment, which could then favor DNA hypermethylation. Therefore, taking into account previous studies we can suggest that hypoxia conditions in the placenta of pregnant women with SCD can induce DNA hypermethylation, and possibly favor placental dysfunctions by the non-differentiation of villous cytotrophoblast to syncytiotrophoblast.

Actually, some differentially methylated genes identified in our study (S3 Table) are associated with hypoxia and stress oxidative, according to the literature.

For the HbSS group, one of the differentially methylated genes identified was a member of the *TGFB* family (*TGFB 1/1)*, which plays important role in the processes of cell growth, proliferation, migration, differentiation, and senescence and can modulate expression and activation of other growth factors. Previous study showed that hypoxia-inducible factor-1 (HIF-1) mediates the biological effects of oxygen on human trophoblastic differentiation through *TGFB* [32]. In pregnancies complicated by early-onset preeclampsia, *TGFB* expression remains abnormally elevated, and trophoblasts are arrested to an intermediate immature phenotype [32]. Moreover, in our study, other differentially methylated genes were found in the HbSS group such as *HNF4A*, *SEPT9* and *GPR56* gene, which also have been reported to be affected by hypoxia [33–35]. As far as we know, this is the first study that associates differential methylation in these genes in the placenta of HbSS patients.

In the HbSC group, among those differentially methylated genes we identified *ROBO2* gene. This gene is involved in several processes, including circulatory system development, neuron development, cell-cell adhesion mediator and identical protein binding. The study conducted by Liao et al. (2012) showed that *ROBO2* gene was downregulated by hypoxia in cells from placenta of pregnant women with preeclampsia and the authors reported that the deregulation of *ROBO2* could impair placental development [36]. In the present study, other differentially methylated genes were found in the HbSC group, and some of them were also reported to be affected by hypoxia, including *TGFB*, *IJAP1* and *GATA4* [37–39].

Oxidative stress plays a critical role in the pathophysiology SCD and can be triggered by the generation of free radicals through release of free hemoglobin, modification of mitochondrial respiratory chain activity and RBC auto-oxidation [40, 41]. It can manifest as multiorgan vasculopathy, which could also affect the placental tissue, as reported in a study on placentas of pregnant women with preeclampsia [42].

Interestingly, in the present study, we identified some differentially methylated genes whose deregulation by oxidative stress has already been reported. Among the genes identified we highlight *LHCGR*, *TGFB1*, *SEPT9* and *BNC2* in the HbSS group [43–46], and *CERS6*, *GATA4*, *HLA*-G and *PAX3* in the HbSC group [47–50]. These findings suggest that oxidative stress may be modulating these genes through methylation in the studied patients.

Therefore, taken together we can suggest that hypoxia and oxidative stress condition, already observed in SCD, may be affecting the methylation status and consequently the expression and function of some genes; however, more studies are needed to confirm this hypothesis.

Additionally, in the present study, both HbSS and HbSC groups showed statistically significant DMPs distribution in the regions related to CpG Island with hypermethylation at CpG Island and S_Shore regions, and hypomethylation at Open Sea region. Approximately 60–70% of the genes in the human genome present CpGs Island, especially in promoter regions, and its methylation is closely associated to gene silencing [51].

Thus, our results indicate that the placenta under SCD conditions can suffer increased methylation in CpG Island regions, which could lead to gene expression alterations in the placental tissue. In this regard, we compared our results with previous studies about DNA methylation in placentas from obese, diabetic and smokers patients. Curiously, the genes found to be differentially methylated in these studies were not present in our methylated gene list, which leads us to hypothesize that HbSS and HbSC patients may have a placental methylation DNA signature induced by the pathophysiology of the hemoglobinopathy. However, further studies are needed to confirm this hypothesis.

The hypermethylation levels were validated in three CpGs sites (cg03949391-*PTGFR*, cg03989617-*GPR56* and cg07274618-*GALR2*) in the HbSS group. Interestingly, two genes presented significant difference in gene expression, being the *PTGFR* with lower expression and the *GPR56* with high expression. Although *PTGFR* has shown a difference in methylation and expression in the HbSS group, we did not observe statistically significant correlation, what can indicate the involvement of other mechanisms of gene expression such as microRNA and/or histone modifications. *PTGFR* encodes a prostaglandin 2α receptor protein, which plays an important role in stimulating trophoblastic cell adhesion, migration and proliferation [52], important events that ensure the appropriate placental function and adequate fetal development [53]. Therefore, we can hypothesize that the lower *PTGFR* expression in HbSS placentas may impair trophoblast function, compromising placental overall function and development. Considering the *GPR56* gene, the correlation analysis revealed a trend for positive correlation, suggesting that hypermethylation may be linked to increased gene expression, what is not common, since methylation has been more associated with decreased gene expression. Lim et al. (2017) demonstrated that, in fact, the correlation between DNA methylation and gene expression in human placenta is more complex than expected [54]. Interestingly, the authors showed a significant correlation between high methylation level at gene body with high gene expression. Hence, we can suggest that the hypermethylation found at the first exon of *GPR56* could be associated with its high expression, nevertheless more studies are necessary to confirm. *GPR56* encodes a membrane receptor protein, which is involved in important pathways such as: cell adhesion, down-regulation of proliferation, cell-cell signaling and brain development. Previous studies in cancer have shown high *GPR56* expression in moderate metastatic tumors, suggesting its role in inhibiting cell proliferation and angiogenesis [55]. Thus, it is possible that increased *GPR56* expression could be inhibiting proliferation and angiogenesis in pregnant HbSS patients, leading to inappropriate development of placental tissue.

For the HbSC group, no CpG site was validated in the pyrosequencing analysis. On the other hand, the expression analysis revealed two differentially expressed genes, being S*POCK1* downregulated and *ADCY4* upregulated. These results can suggest that other regulatory mechanisms as microRNA and/or histone modification can be involved in their altered expression other than DNA methylation, but more studies need to be performed to demonstrate this hypothesis.

Regarding *SPOCK1*, *s*tudies have demonstrated that it plays a critical role in cell proliferation, invasion and migration in different types of tumors [56–58]. A recent study has shown that the *SPOCK1* silencing reduced the migration capacity of colorectal cancer cell lines, [56], supporting our hypotheses that low *SPOCK1* expression in the placenta of HbSC pregnancies may impair the migration, invasion and cell proliferation mechanisms, which could compromise the placental function.

Considering the *ADCY4* gene, the findings showed a positive correlation between methylation in the promoter region and increased expression. Most of the times methylation in promoter regions is associated with reduced gene expression, however there are studies on various types of cancer that report a positive correlation between methylation located in promoter regions and the increased gene activity [59, 60]. As an example, a study in colon cancer has reported high methylation level in the promoter region and it was positively correlated with gene expression [61]. However, the mechanism by which methylation in promoter regions may increase gene activity is still poorly understood. The A*DCY4* gene encodes a member of the family of adenylate cyclases that are membrane-associated enzymes, responsible for physiological changes, such as: cell control, differentiation, vesicle translocation, enzyme production and apoptosis [62]. A recent study on gene expression in human placenta reported increased expression of the *ADCY4* gene in the first trimester compared to the third trimester, indicating that pathways related to this gene may be more active in early pregnancy [63]. Thereby, due to the increased *ADCY4* expression in placentas from third trimester (HbSC group), we suggest that biological processes involved *ADCY4* could be increased in this gestational age, impairing the placenta development in women with SC hemoglobinopathy.

In our study, the presence of adverse maternal and perinatal outcomes confirmed the increased morbidity associated with SCD, which included lower gestational age at birth, especially in the HbSS genotype, high prevalence of preterm birth and lower maternal body mass index. SCD cases also showed significant differences in placental weight and birth weight, which are also mostly associated with the differences in gestational age at delivery.

In the present study, the methylation analysis was evaluated from the layer of chorionic villous in the placenta tissue. This layer is composed by a distinct cell types, such as mesenchymal cells, endothelial cells, blood cells, macrophages, myofibroblasts, smooth muscle cells and fibroblasts [64]. We did not perform any cell culture to isolate a specific cell type. We acknowledge such limitation, once that different cells might show specific methylation profile. However, the sampling method was very consistent throughout considered cases, following a protocol available in the literature for this type of sampling. Although our study has some limitations, including the multicellularity in villous tissues, small sample size and the different gestational age in the groups, to the best of our knowledge, it is the first study that has evaluated the DNA methylation profile in placentas from pregnant women in the two most frequent genotypes of SCD (HbSS and HbSC).

In conclusion, our findings suggest that SCD may affect placental DNA methylation, leading to a hypermethylation status. This increased methylation may lead to changes in placental gene expression and consequently disrupt trophoblast function, contributing to inadequate placental development. Our results have provided new insights that may head future research towards a better understanding of the mechanisms and pathways underlying DNA methylation involvement in the epigenetic regulation of major placental processes in pregnant women with SCD.

## Supporting information

S1 Table Selected DMRs for methylation validation. (DOCX)

S2 Table DMPs list and methylation beta-value for each patient in the HbSS, HbSC and Control groups. (XLSX)

S3 Table DMRs identified from the comparison between HbSS and HbSC groups versus control group. (XLSX)

S4 Table Data of gene expression levels for each studied cohort. (XLSX)
