# Supplementary material for: Epigenetic analysis in placentas from sickle cell disease patients reveals a hypermethylation profile
Source: PLoS One. 2022 Sep 21;17(9):e0274762. doi: 10.1371/journal.pone.0274762 (PMC9491616; doi:10.1371/journal.pone.0274762)
Supplement: S1 Table — (DOCX) [file pone.0274762.s001.docx]

**S1-Table**. Selected DMRs for methylation validation. The probes (CpGs sites) submitted to methylation analysis by pyrosequencing are in bold.

| **Study group** | **DMRs – coordinates** | **Δ-β (methylation status)** | **length (pb)** | **Probes** | **CpG location (gene)** | **CpG location (CpG Island)** | **Gene** | **Adjusted**  **p-value** |
| --- | --- | --- | --- | --- | --- | --- | --- | --- |
|  |  |  |  |  |  |  |  |  |
| HbSS | chr1:78956576-78956905 | 0.2  (hypermethylation) | 330 | cg03842686 | TSS200 | Island | *PTGFR* | <0.001 |
|  |  |  |  | cg20092458 | TSS200 | Island |  |  |
|  |  |  |  | **cg03949391** | 1stExon | Island |  |  |
|  |  |  |  | cg27046936 | 1stExon | Island |  |  |
|  |  |  |  | cg03495868 | 1stExon | Island |  |  |
|  | chr16:57662541-57662690 | 0.115 (hypermethylation) | 150 | **cg03989617** | 1stExon | OpenSea | *GPR56* | 0.007 |
|  |  |  |  | cg09730500 | 1stExon | OpenSea |  |  |
|  | chr17:74070375-74070698 | 0.192 (hypermethylation) | 324 | cg10854758 | TSS1500 | S_Shore | *GALR2* | <0.001 |
|  |  |  |  | cg27494087 | TSS1500 | Island |  |  |
|  |  |  |  | cg16898239 | TSS1500 | Island |  |  |
|  |  |  |  | **cg07274618** | TSS200 | Island |  |  |
|  | chr14:24803679-24803873 | 0.171 (hypermethylation) | 194 | cg07485357 | Body | Island | *ADCY4^#^* | <0.001 |
|  |  |  |  | **cg23179456** | TSS200 | Island |  |  |
| HbSC | chr5:136834383-136834464 | 0.165 (hypermethylation) | 82 | cg20897685 | 5'UTR | Island | *SPOCK1* | 0.0101 |
|  |  |  |  | **cg24847829** | 5'UTR | Island |  |  |
|  | chr7:11871535-11872050 | 0.186 (hypermethylation) | 516 | cg26616283 | 1stExon | OpenSea | *THSD7A* | <0.001 |
|  |  |  |  | cg26748945 | 1stExon | OpenSea |  |  |
|  |  |  |  | cg12348203 | TSS200 | OpenSea |  |  |
|  |  |  |  | cg17230649 | TSS200 | OpenSea |  |  |
|  |  |  |  | **cg24676244** | TSS1500 | OpenSea |  |  |
|  | chr14:24803679-24804339 | 0.158 (hypermethylation) | 661 | cg07485357 | Body | Island | *ADCY4^#^* | 0.0103 |
|  |  |  |  | **cg23179456** | TSS200 | Island |  |  |
|  |  |  |  | cg13631572 | TSS200 | Island |  |  |
|  |  |  |  | cg25556905 | TSS200 | Island |  |  |
|  |  |  |  | cg14287235 | TSS1500 | Island |  |  |

Δ-β: methylation difference between cases (HbSS or HbSC) and controls, **#** analyzed in HbSS and HbSC groups.

**CpG location (gene)**: TSS1500: up to 1500 bases from the transcription start site (TSS), TSS200: up to 200 bases from the transcription start site, 5' and 3' UTR: untranslated region, first exon, gene body or outside of this region classified as intergenic region;

**CpG location (CpG Island)**: S Shore: up to 2000 bases South of CpG island, N Shore: up to 2000 bases North of CpG island, S Shelf: from 2000 to 4000 bases South of CpG island, N Shelf: from 2000 to 4000 bases North of CpG island, Open Sea: over 4000 bases from CpG Island.
